# Spatial surveillance of *Plasmodium falciparum* Kelch 13 mutations reveals High-Frequency A675V and the First Molecular Evidence of Suspected Partial Artemisinin Resistance in Baringo in Kenya

**DOI:** 10.1186/s12936-026-06102-9

**Published:** 2026-10-05

**Authors:** Hoseah M. Akala, Benjamin H. Opot, Dennis W. Juma, Raphael O. Okoth, Maurine Mwalo, Joseph G. Amwoma, Farid A. Salim, Risper Maisiba, Redemptah Yeda, Edwin W. Mwakio, Gladys Chemwor, Jackline A. Juma, Charles O. Okudo, Timothy E. Egbo, Doris Njoroge, Michal M. Ohaga, Agnes C. Cheruiyot, Regina Kandie, Kibor Keitany, Sammy Mahugu, Kristan A. Schneider, Victor Osoti, Kevin Wamae, Milton A. Obilo, George K. Ng’ang’a, Eric C. Garges, Ben Andagalu, Edwin Kamau, Lynette Isabella Ochola-Oyier, Bernhards R. Ogutu

**Affiliations:** 1 Kenya Medical Research Institute (KEMRI)/Walter Reed Army Institute of Research - Africa, Kisumu, Kenya; 2 Walter Reed Army Institute of Research - Africa, Nairobi, Kenya; 3 Department of Pathology and Area Laboratory Services, Tripler Army Medical Center, Honolulu, HI USA; 4 Biosciences Department, KEMRI-Wellcome Trust Research Programme, Kilifi, Kenya; 5 University of New Mexico School of Medicine, Albuquerque, NM, USA; 6 Kenya Ministry of Defense, Nairobi, Kenya; 7 Center for Clinical Research, Kenya Medical Research Institute, Nairobi, Kenya; 8 Division of National Malaria Programme, Ministry of Health, Nairobi, Kenya; 9 Department of Emerging and Infectious Diseases, Walter Reed Army Institute of Research-Africa/Centre for Clinical Research, Kenya Medical Research Institute, P. O. Box 54, Kisumu, 40100 Kenya

## Abstract

**Background:**

This surveillance study evaluated the polymorphisms of *Pfk13* gene in samples from symptomatic individuals from eight geographically distinct locations in Kenya to determine the prevalence of mutations associated with partial artemisinin resistance.

**Methods:**

Between 2018 and 2024, blood samples from individuals with symptoms of uncomplicated malaria at eight hospitals in four of the five distinct malaria transmission zones of Kenya were sequenced for single-nucleotide polymorphisms (SNPs) in *Pfk13* and *Pfmdr1*. Subsequent positive samples were tested to monitor treatment outcomes.

**Results:**

A total of 44/675 (3.0%) samples harbored 49 *Pfk13* mutations. These include 14 nonsynonymous mutations at *Pfk13:* A675V (*n* =  3.0%) A578S (*n* = 4, 0.6%), C469Y (*n* = 3, 0.44%), V386A (*n* = 1, 0.15%), P553L (*n* = 1, 0.15%), R561H/P (*n* = 1, 0.15%), S522C (n = 1, 0.15%), K455E (*n* = 1, 0.15%), S600F (*n* = 1, 0.15%), E612D (*n* = 1, 0.15%), N489K (n = 1, 0.15%), F491L (*n* = 1, 0.15%) plus A504V (*n* = 1, 0.15%). Four WHO-validated markers of partial artemisinin resistance were detected in 25/675 (3.7%) samples. Most Pfk13 A675V mutations (n = 12, 1.8%), constituting 60% of all Pfk13 mutations observed by this study were detected in Baringo County, where the mutation frequency reached 12/71 (16.9%), exceeding the WHO 5% threshold for classification of areas with suspected partial artemisinin resistance. This represents the first molecular evidence consistent with suspected artemisinin resistance in Baringo County.

**Conclusion:**

The detection of four WHO-validated Pfk13 markers of partial artemisinin resistance across multiple malaria transmission zones indicates emerging artemisinin resistance in Kenya. The high frequency of A675V in Baringo provides the first molecular evidence consistent with suspected partial artemisinin resistance in Kenya and highlights the need for enhanced therapeutic efficacy, deployment of Multiple First-line Therapies, and molecular surveillance.

**Supplementary Information:**

The online version contains supplementary material available at https://doi.org/10.1186/s12936-026-06102-9.

## Introduction

Confirmed reports of artemisinin tolerance in Southeast Asia (SEA) threaten the continued use of artemisinin-based combination therapies (ACTs) for the treatment of uncomplicated malaria globally [1, 2]. Studies show that mutations in the *Plasmodium falciparum* Kelch 13 gene (*Pfk13*) associated with delayed parasite clearance in vitro and in vivo [3] are widespread in SEA [4, 5]. The first cases of *Pfk13* mutations-associated with artemisinin partial resistance were identified in 2013 in Africa. Initially reported in Rwanda and Ethiopia, these mutations were not associated to those previously detected in SEA [6, 7]. Subsequently, they were being detected in Eritrea [8], Tanzania [9] and Kenya [10, 11]. Specifically, artemisinin delayed parasite clearance in Africa has been linked to the *Pfk13* 622I mutation in Ethiopia, the R561H mutation, which is undergoing clonal proliferation southwards in Rwanda [7, 12] into northern Tanzania [9], and the C469Y and A675V mutations in Eastern Uganda [13] and western Kenya [10, 11]. The World Health Organization (WHO) classifies regions where validated or candidate molecular markers of partial artemisinin resistance exceed the recommended thresholds of 5% as areas with suspected partial artemisinin resistance, whereas regions with confirmed clinical evidence of delayed parasite clearance exceeding 5% are classified as confirmed artemisinin resistance areas.

The most widely studied and well characterized ACTs in Africa are artemether-lumefantrine (AL), artesunate-amodiaquine (ASAQ), dihydroartemisinin-piperaquine (DHAPPQ), artesunate-mefloquine (ASMQ), and artesunate-pyronaridine [14]. Of these, AL is the most widely used treatment in Africa. The nonsynonymous mutations N86Y, Y184F, S1034C, N1042D within the *Pfmdr1* gene have been implicated in modulating parasite response to chloroquine and ACTs partner drugs amodiaquine, lumefantrine, and mefloquine [15]. Notably, AL and ASAQ exert inverse selective pressures whereby parasites with genotype 86Y, Y184 and 1246Y are selected by ASAQ treatment, N86, 184F, and D1246 are selected by AL treatment [15]. Since the strategy behind the deployment of ACTs relies on the slow acting partner drug to clear the residual parasitemia after initial rapid clearance of most asexual parasites by the short half-life, fast-acting artemisinin component, the delayed or partial parasite clearance by the artemisinin derivative will result in prolonged exposure of the parasite to the partner drug. This therefore renders the partner drug vulnerable to rapid development of resistance as a major concern for the combination therapy approach. Owing to initial reports in southeast Asia [1], the emergence of resistance to artemisinin and partner drugs has been identified as a significant risk to the global effort to reduce the malaria burden as per the Global Technical Strategy (GTS) for malaria [16], necessitating intensified surveillance for the early detection of resistance.

This surveillance study sought to determine the frequency of polymorphisms in *Pf*k13 gene, and Pfmdr1 gene focusing on markers of malaria drug resistance from clinical samples collected in eight hospitals located in geographically diverse regions across Kenya within four of the five malaria transmission zones of Kenya between 2018 and 2024. This study identified nonsynonymous mutations across 14 *Pfk13* codons, including four validated markers of partial artemisinin resistance present in 25/675 (3.7%) samples. Together these findings indicate active genomic diversification at the *Pfk13* locus consistent with emerging artemisinin resistance in Kenya, posing a potential threat to the continued effectiveness of artemether–lumefantrine for the treatment of uncomplicated malaria.

## Methods

### Study sites and sample collection

Between July 2018 and June 2024, we conducted a longitudinal study to monitor temporal trends in *Plasmodium species* prevalence and antimalarial resistance markers across eight health facilities located in seven Kenyan counties spanning across four of the five malaria transmission zones comprising three sites in the Lake endemic zone (Busia–Busia County Referral Hospital; Kisumu–Kombewa Sub-County Hospital; and Kisumu County Referral Hospital), two sites in the highland zone (Kisii–Kisii County Referral Hospital; Kericho–Kericho County Referral Hospital), semi-arid seasonal transmission zone (Baringo–Marigat Sub-County Hospital) as well as low risk zone (Nakuru–Lanet Regional Hospital; Laikipia–Laikipia Hospital). These sites were chosen to span four of the five distinct malaria transmission zones across Kenya National Malaria Control Programme 2020 [17, 18]. Throughout the year, study personnel residing at the study’s sentinel sites situated within the outpatient department recruited participants among patients presenting to outpatient departments with uncomplicated malaria, who were willing to participate in the study. Patients aged six months and older presenting to the outpatient departments and diagnosed with uncomplicated malaria by rapid diagnostic test (mRDT; Parascreen® (Pan/Pf), Zephyr Biomedicals, Verna Goa, India) were recruited into the study after providing written informed consent or assent. Pregnant women were also eligible for enrollment. Individuals were excluded from the study upon refusal to participate or unwillingness to give blood. The study also excluded prisoners, including children attending the Kenyan government’s correction and rehabilitation programme. Additionally, individuals under age 18 without available parent or legal guardian, volunteers who were previously enrolled in the study during the same calendar year, individuals weighing less than 5 kg, and anyone who, in the opinion of the attending medical provider, would be adversely affected by the drawing of 2.5 mL of blood were excluded. Written informed assent for individuals aged 17 years and below was provided by their parent or legal guardian in accordance with the laws of the government of Kenya. Individuals who did not meet the inclusion criteria were excluded as previously described [19]. For all consenting and assenting individuals who were enrolled in the study, 5 ml of whole blood was collected, and aliquots of 1.5 mL were dispensed in the Ethylenediaminetetraacetic acid tube for molecular analyses, while the remainder was used for other endpoints of the study. The sample was swirled well in the anticoagulant to homogeneity, retained at room temperature for 30 min, then stored at 2 to 8 °C until transportation at the same temperature to the central lab for processing. It is noteworthy that consecutive laboratory-confirmed malaria cases were enrolled at sentinel sites. Sample numbers reflected local malaria burden and case availability, resulting in unequal sample sizes across sites.

### Case management

For case management, all *Plasmodium* positive cases were treated with Coartem® (Novartis, Basel, Switzerland), which contains artemether-lumefantrine (AL) in accordance with the Kenya Ministry of Health recommended case management guidelines for uncomplicated malaria. After drawing blood, the attending clinician administered and observed the first dose of Coartem® 20 mg/120 mg tablets, with individuals weighing 5 to < 15 kg prescribed 1 tablet per dose, 15 to < 25 kg 2 tablets per dose, 25 to < 35 kg 3 tablets per dose and individuals greater than 35 kg 4 tablets per dose. Each patient was given the remaining 5 doses (i.e. 5, 10, 15, 20, and 25 tablets for each weight range) and advised to take the next dose after eight hours, followed by the remaining doses at 12 hourly intervals until completion of treatment.

Furthermore, each participant was required to come back on day 7 for clinical and laboratory evaluation, comprising documentation of vital signs as well as parasitological diagnosis of malaria. Regardless of the outcomes of these evaluations, an additional blood draw was taken during day 7 visit or any other visit day within 42 days follow-up for molecular and vitro testing at the central lab to monitor treatment outcomes. Importantly, all participants were encouraged to come back to the study clinician on any other day during the subsequent 42 days for review and further clinical management symptoms persist. This study was conducted under the approval of the Kenya Medical Research Institute (KEMRI), Scientific and Ethics Review Unit (SERU) and Walter Reed Army Institute of Research (WRAIR) institutional review boards, protocol numbers: WRAIR 2454, KEMRI 3628: “Epidemiology of malaria and drug sensitivity patterns in Kenya.”

### Parasite density estimation

Three glass microscope slides for thick and thin smears were prepared from the whole blood sample obtained from each participant. One slide was read the same day and reported, the two remaining slides were batched and read at a later date. All positive *Plasmodium* infections were characterized for parasitemia. Parasites were typed counts per number of cells counted in either 200 or 2000 high power fields for thick and thin malaria blood smears, respectively, before expressing as either percent parasitemia or parasite density, as previously described [20]. Parasites per number of white blood cells were counted on thick blood smears, whereas parasites per number of red blood cells were counted on thin blood smears.

### Plasmodium falciparum DNA extraction and sequencing

DNA extraction from whole blood was done using Qiagen DNA Mini extraction spin protocol (Qiagen, Valencia, CA) as per the manufacturer’s guidelines and eluted with 150 µL of elution buffer. Molecular diagnosis of *Plasmodium* genus and species was performed using a quantitative real-time PCR using primers targeting 18S ribosomal RNA as described elsewhere [21]. This procedure involved two reactions. The first detected the presence of *Plasmodium* genus using the real-time PCR assay on Applied Biosystem QuantStudio 6 flex (QuantStudio™ real-time PCR Applied Biosystems by Thermos Fisher Scientific Foster City, CA) as previously described [21, 22]. The second reaction on the same platform, using the assay conditions and primers previously described [23, 24], identified the *Plasmodium* species present in the infection. Samples that were positive for the *Plasmodium falciparum* species with cycle threshold values of 31 and below were further processed for *Pfk13* and mutation analysis. MSP1/2 and GLURP genotyping was conducted for all samples that tested positive for *P. falciparum* by PCR during the 42-day follow-up period to assess for recrudescent infections.

### Single nucleotide polymorphisms in the *Pfk13,* and *Pfmdr1* genes

A conventional PCR was performed using primers as described by de Laurent et al. [25] to amplify the *Pfk13* and *Pfmdr1,* genes for sequencing. This resulted in a detectable band size consistent with successful amplification of the *Pfk13, and Pfmdr1* genes. Exo-SAP IT was used to purify the PCR products by removing excess nucleotides and primer remnants. Sanger sequencing was performed on purified PCR products using Big Dye terminator method version 3.1 on the 3500xL ABI genetic analyzer (Applied Biosystems, Foster City, CA). Sequencing data was then analyzed on the Qiagen CLC Main Workbench (Qiagen, Valencia, CA) (Supplementary Table 1).

### Sequencing of the *Pf1k13*, and *Pfmdr1* genes amplicons

#### Library preparation and sequencing

A total of 927 isolates were subjected to amplicon sequencing. The amplicons were purified using Exo-SAP IT to remove primers and other short DNA fragments following manufacturer’s instructions. These amplicons were then normalized to a concentration of 2 ng/µL for DNA library preparation. Libraries were generated using the Nextera XT library preparation kit, following the manufacturer’s protocol. Using Tape Station 4200 system (Agilent Technologies, Santa Clara, CA, USA) D1000 screen Tape was used to confirm adaptor ligation. The libraries were pooled for sequencing on Illumina iSeq 100 (Illumina Inc, San Diego, CA). Libraries were sequenced on the Illumina MiSeq platform, and variants were called only in regions with a minimum read depth of 30 and a per-base Phred quality score ≥ 30. Samples failing to meet either criterion for ≥ 90% of target amplicons were excluded from downstream NGS analyses (Supplementary Table 1).

#### Sequencing data processing

Data processing was performed as previously described by Niaré and Bailey [26]. Microhaplotype clustering was performed using the SeekDeep v3.0.0 workflows as previously described [27]. GATK4 Haplotype Caller was also used to call variants with the following parameters described by [26]).

Using BCFtools, SNPs with a DP > 100, minor allele frequency (MAF) < 0.02, and > 25% missingness were excluded. Alleles with a frequency below 0.5 were considered minor alleles, while those above were considered major alleles. Functional annotation of the SNPs was performed using SnpEff annotation tool.

Haplotypes constructions were conducted by aligning PCR-amplified sequences to a reference genome using ClustalW to identify SNPs relative to the wildtype. After variant calling, we applied stringent quality control measures—including filters based on minor allele frequency and Hardy–Weinberg equilibrium—to ensure data reliability. The resulting SNP dataset was then processed using a Bayesian algorithm to infer haplotype phases, enabling the accurate assembly of both single and multiple mutation haplotypes. Finally, haplotype frequencies were determined by calculating the proportion of each haplotype relative to the total number of sequences analyzed.

### Results mapping

Mutation mapping was achieved by compiling mutation data in Excel and mapping it onto an OpenStreetMap (OSM) basemap using Quantum GIS (QGIS), a free and open-source Geographic Information System (GIS) software was used for mapping, spatial data analysis, and geospatial visualization. QGIS allowed overlaying datasets onto basemaps while applying various spatial analysis techniques. The final map was then exported as an image.

### Data management and statistical analyses

Data were initially collected on standardized case report forms and subsequently entered into a secure, dedicated database. To ensure data integrity, we implemented double data entry, performed regular quality checks, and conducted range and consistency validations. For statistical analysis, categorical data, including gene sequence polymorphisms were summarized as frequencies and proportions.

Raw data files will be available upon request, with the eventual intention of depositing all sequence data on the NCBI platform for unrestricted public access.

## Results

**Fig. 1:**
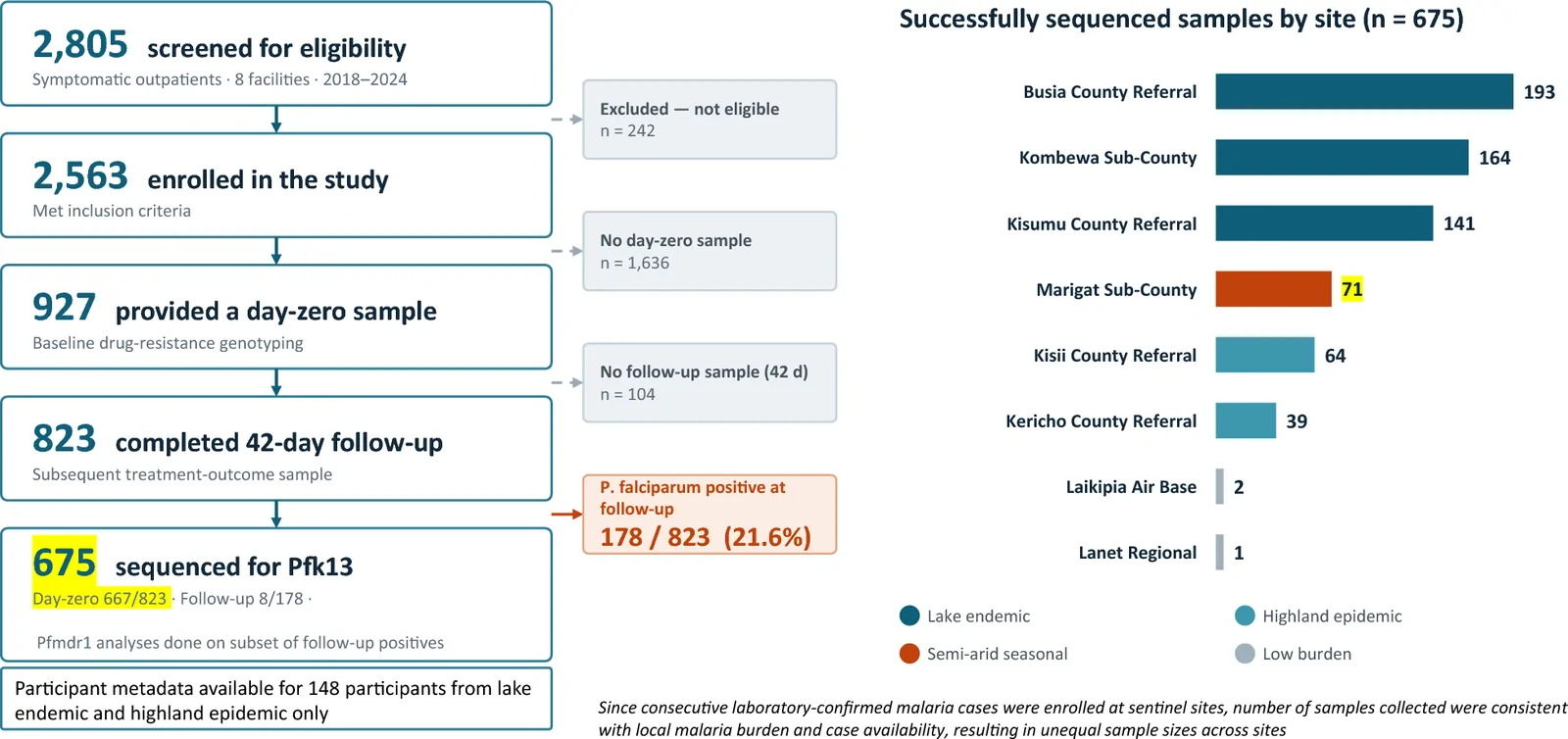
Participant flow and sequenced samples for Symptomatic outpatients at 8 sentinel sites July 2018–June 2024

Between July 2018 and June 2024, 2,805 study participants consisting of symptomatic patients presenting at the outpatient departments of eight healthcare facilities were screened, of which 2,563 met inclusion criteria and were enrolled in the epidemiology of malaria drug resistance surveillance study. Of the 927/2563 participants that provided day-zero samples, 823 were followed up successfully giving a second subsequent sample during the 42-day follow-up period for treatment outcome monitoring. Of the individuals with a subsequent sampling timepoint within the 42-day follow-up period, 178/823 (21.6%) individuals tested positive for *P. falciparum.* Of these, 667/823 day zero and 8/178 subsequent follow-up days samples were successfully sequenced for polymorphisms in Pfk13. A subset of samples that tested positive during the follow-up period were characterized for their day zero Pfmdr1 polymorphisms. The successfully sequenced samples comprised those from hospitals namely Busia County Referral (*n* = 193), Kombewa Sub-County *n* = 164, Kisumu County Referral *n* = 141 both in Kisumu County, Kisii County Referral (*n* = 64) Kericho County Referral (*n* = 39), Nakuru county at Lanet Regional (*n* = 1), Laikipia county’s Laikipia Air Base (*n* = 2), Baringo county’s Marigat Sub-County (*n* = 71) hospitals (Fig. 1). Parasitemia data were available for 673 of 675 samples with complete *Pf*k 13 sequence data. The median parasitemia and interquartile range was 0.70% [0.15–2.60; n = 673 of the sequenced isolates]. Per-county median parasitemia and interquartile range were Kericho 2.30% [0.40–7.00, n = 37]; Kisumu 1.55% [0.18–4.00, n = 304]; Busia 0.52% [0.17–1.60, n = 191]; Baringo 0.60% [0.08–1.50, n = 74]; and Kisii 0.20% [0.10–0.27, n = 64]. The low-burden counties contributed one sample from Nakuru (1.00%) and two from Laikipia (0.04% and 0.20%). Parasite density was not available for six sequenced samples (Supplementary Table 3). Since consecutive laboratory-confirmed malaria cases were enrolled at sentinel sites, number of samples collected were consistent with local malaria burden and case availability, resulting in unequal sample sizes across sites. Complete demographic metadata were available for 148 participants who had complete case report forms. Accordingly, Table 1 summarizes only participants with complete demographic data therefore excluding participants from the semi-arid seasonal and low burden transmission regions Table 1. Analysis of these participants showed that those from the participants from the Lake endemic region were younger and reported more malaria episodes during the preceding 12 months than those from the highland epidemic region.

**Table 1:** Study site demographics and clinical summaries

Site (n)	Region^a^	Age in years*	Sex (% Female)	Temperature in degrees Celsius*	Malaria episodes in last 12 months
Busia (61)	Lake Endemic	11.38 [7.72–15.04] (60/61)	60.66% (37/61)	37.77 [37.50–38.04] (60/61)	37/61
Kisumu (13)	Lake Endemic	11.85 [4.04–19.65] (13/13)	61.54% (8/13)	38.29 [37.66–38.92] (13/13)	6/13
Kombewa (32)	Lake Endemic	10.96 [8.10–13.82] (27/32)	59.38% (19/32)	37.77 [37.46–38.09] (32/32)	25/27
Kisii (27)	Highland Epidemic	13.38 [7.54–19.22] (26/27)	37.04% (10/27)	38.73 [38.30–39.17] (26/27)	8/26
Kericho (15)	Highland Epidemic	23.46[17.17–29.75] (15/15)	46.67% (7/15)	37.67 [36.88–38.47] (15/15)	2/15
All (148)		13 [10.81–15.19] (141/148)	54.73% (81/148)	37.98 [37.79–38.16] (146/148)	78/142

*Mean [95% Confidence Interval] (n/N). Region^a^ refers to the transmission zone based on Kenya’s malaria burden map as per the Kenya National Malaria Indicator Survey 2020 (KIMS 2020)

### *Plasmodium falciparum* single nucleotide polymorphism in *Plasmodium falciparum* Kelch 13 gene

A total of 44 distinct mutations in *Pfk13* gene were detected in 675 samples using Sanger and amplicon sequencing techniques (Table 2) and a subset confirmed by NGS using iSeq100 Sequencing System—Illumina. Samples that did not meet the inclusion threshold due to low depth were excluded. Those included in this study report comprised 13 nonsynonymous mutations in 36 samples including C469Y *n* = 3, (0.5%), P553L *n* = 1, (0.15%), R561H *n* = 1 (0.15%) and A675V *n* = 20 (2.9%) that are validated markers of partial artemisinin resistance. Other non-synonymous mutations included A578S *n* = 4 (0.6%) as well as V386A, R561H/P, S522C, K455E, S600F, E612D, N489K, F491L plus A504V at *n* = 1 (0.15%). A total of 11 synonymous mutations were also detected in 13 samples (Table 1). Notably, seven of the nonsynonymous and six of the synonymous mutations had not previously been reported elsewhere. One infection from Kisii had triple *Pfk13* G497G/P553L/L488L mutation while two infections from Busia had double *Pfk13* mutations, A675V/V386V and A675V/ C469Y (Table 1). A total of 21 out of the 24 participants who harbored validated partial artemisinin resistance mutations returned on day 7. All tested negative by microscopy with five showing late amplifications cycle thresholds but failing to amplify for recrudescence reinfection analyses.

**Table 2:**
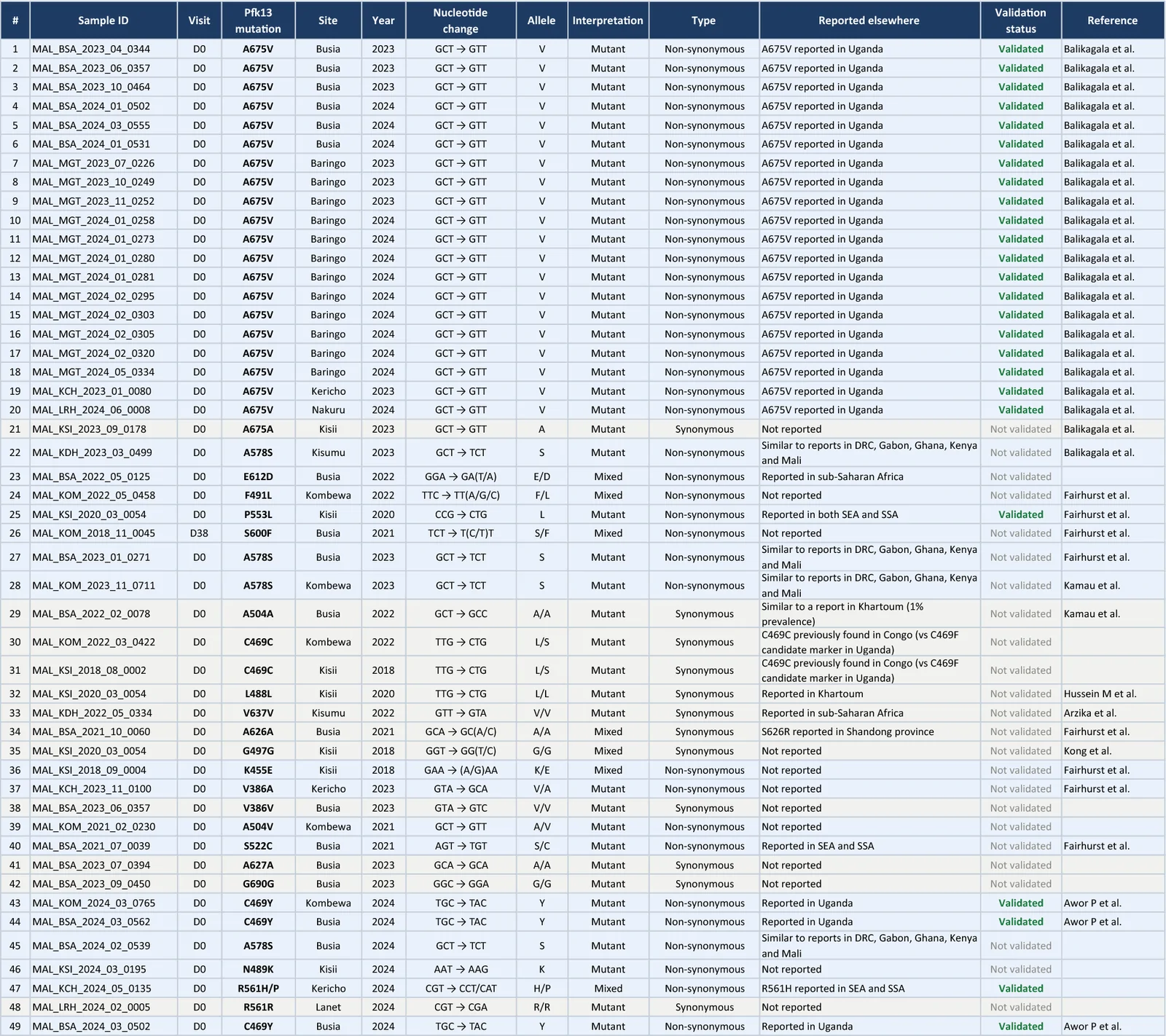
Synonymous and non-synonymous polymorphisms in *Plasmodium falciparum Pfk13* gene in field isolates

Colour: blue rows = non-synonymous polymorphisms; grey rows = synonymous. Bold green "Validated" = WHO-validated marker of partial artemisinin resistance

The list comprises 49 polymorphisms detected in 44 isolates: 25 validated markers (A675V ×20, C469Y ×3, P553L ×1, R561H/P ×1) and 24 other polymorphisms. Isolate MAL_KSI_2020_03_0054 carries three polymorphisms (P553L/L488L/G497G)

### Increasing frequency of *Pfk13 A675V*

**Fig. 2:**
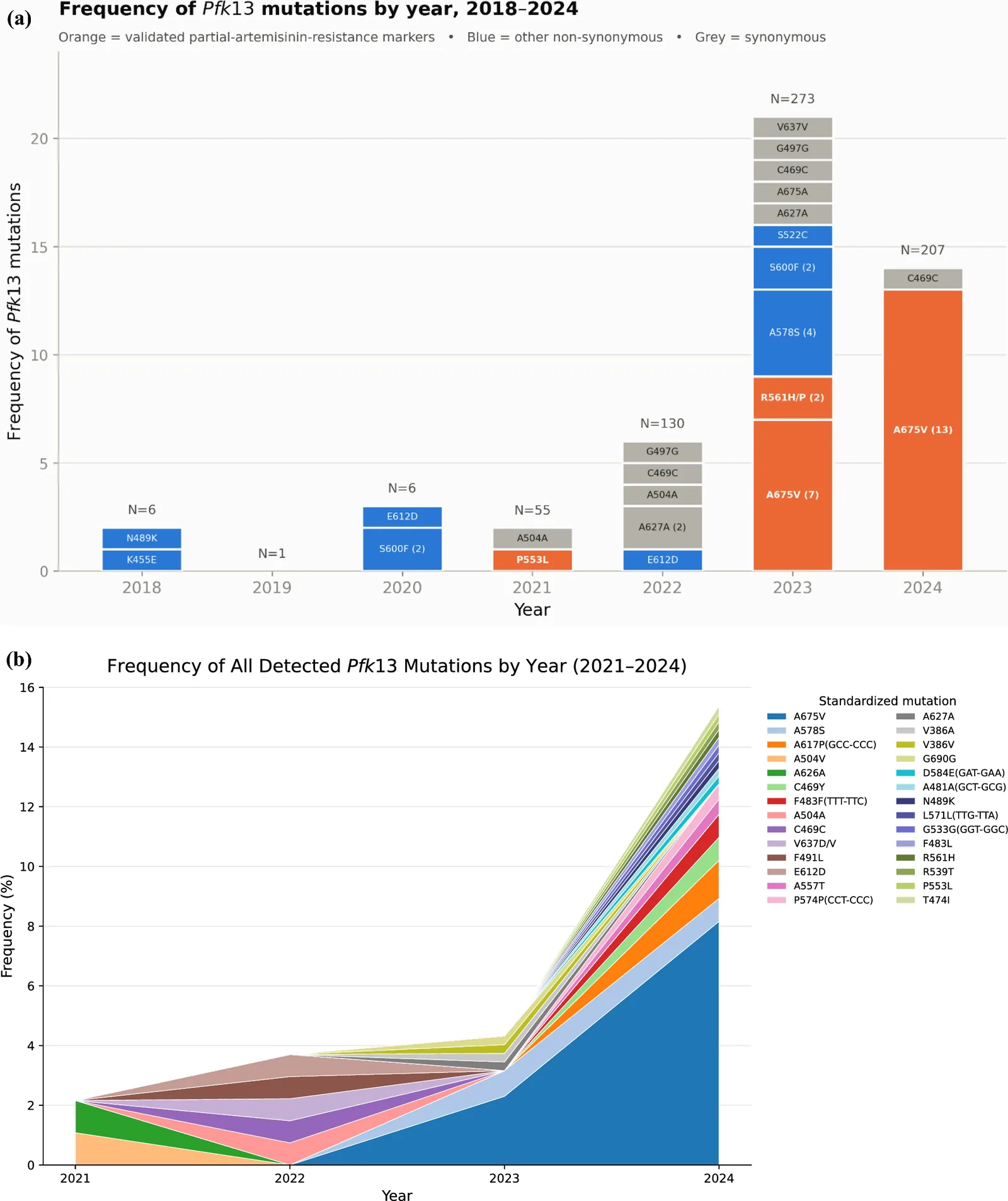
**a**, **b** Frequency* of Pfk13* mutations between 2018 and 2024

An increase in mutation frequency was observed in 2023 for A675V (Fig. 2a, b). Temporally, *Pfk13* 675 V was the first mutation to be detected in two samples from Busia and Kericho between January and June 2023. By December 2023, the mutation frequency increased by six additional samples, followed by an additional 14 by June 2024 bringing the frequency of this mutation to 3.1%, (Fig. 2a, b).

### Geographical distribution

**Fig. 3:**
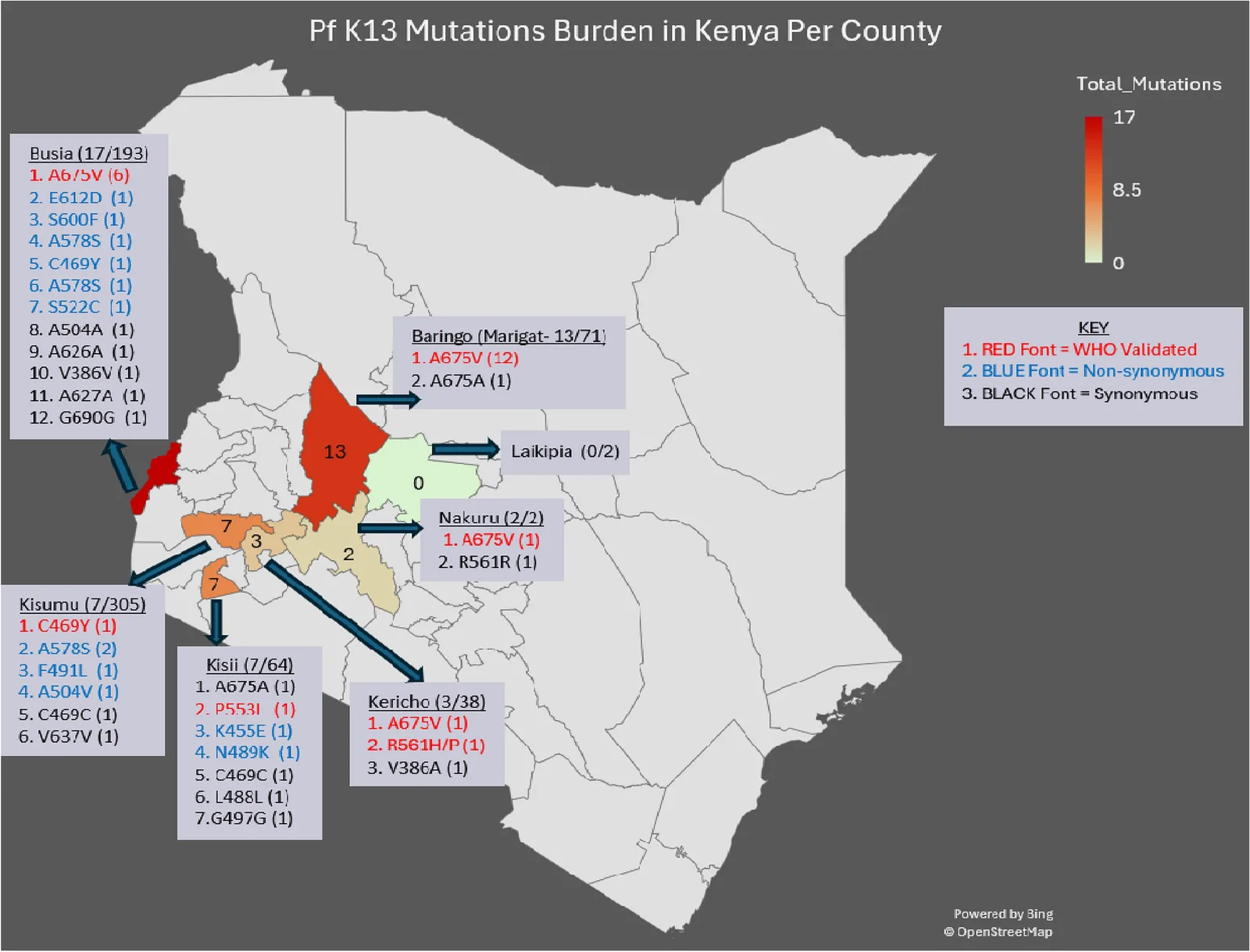
Map of Kenya showing the geographic distribution of *Pfk13* mutations between 2018 and 2024. Color difference represents the relative frequency of validated artemisinin mutations for each County. Highest frequency in Baringo followed by Busia. Single nucleotide polymorphisms font red = validated, blue = nonsynonymous, black = synonymous mutations. A675A in Baringo represents a base change

Geographically, the *Pfk13* alleles were distributed among the seven study sites where Busia had the highest number of allele variants with 17 different alleles present in 15 samples followed by Kisii with seven different alleles in five samples while Baringo County (Marigat) had the least allele frequency with one allele in all the 12 samples sample that were polymorphic (Table 3, Fig. 3). The WHO validated partial artemisinin resistance allele A675V was the most prevalent, occurring at 3.0% (*n* = 20/675) of the infections followed by A578S at 0.6% (*n* = 4), C469Y at 0.45% (*n* = 3) while R561H, were at 0.15% (n = 1) (Table 1). Synonymous mutation C469C, was detected in two samples obtained from Kisii and Kisumu (Table 1). Seven nonsynonymous and six synonymous mutations had not been reported previously. Assessment of the haplotype frequency is summarized in supplementary Table 3.

**Table 3:** Frequency and distribution of *Pfk13* mutation and samples in the eight study sites

County	Study site	Non-synonymous mutation	Synonymous mutation	Alleles (N = 49)	Frequency per site
Busia	Busia CRH	12	5	17 (34.7%)	17/193 (8.8%)
Kisumu	Kombewa Sub-CRH and Kisumu CRH	6	2	8 (16.3%)	8/305 (2.6%)
Kericho	Kericho CRH	3	0	3 (6.1%)	3/39 (7.7%)
Kisii	Kisii CRH	3	4	7 (14.3%)	7/64 (10.9%)
Baringo	Marigat Sub-CRH	12	1	13 (24.5%)	13/71 (18.3%)
Nakuru	Lanet Regional Hospital	1	1	2 (4.1%)	2/4 (50%) *
Laikipia	Laikipia Air Base Hospital	0	0	0 (0)	0/2 (0%) *

CRH means County Referral Hospital, * High percentage due to the low number of samples analyzed from the county given the low malaria endemicity in the region despite lengthy surveillance duration

**Fig. 4:**
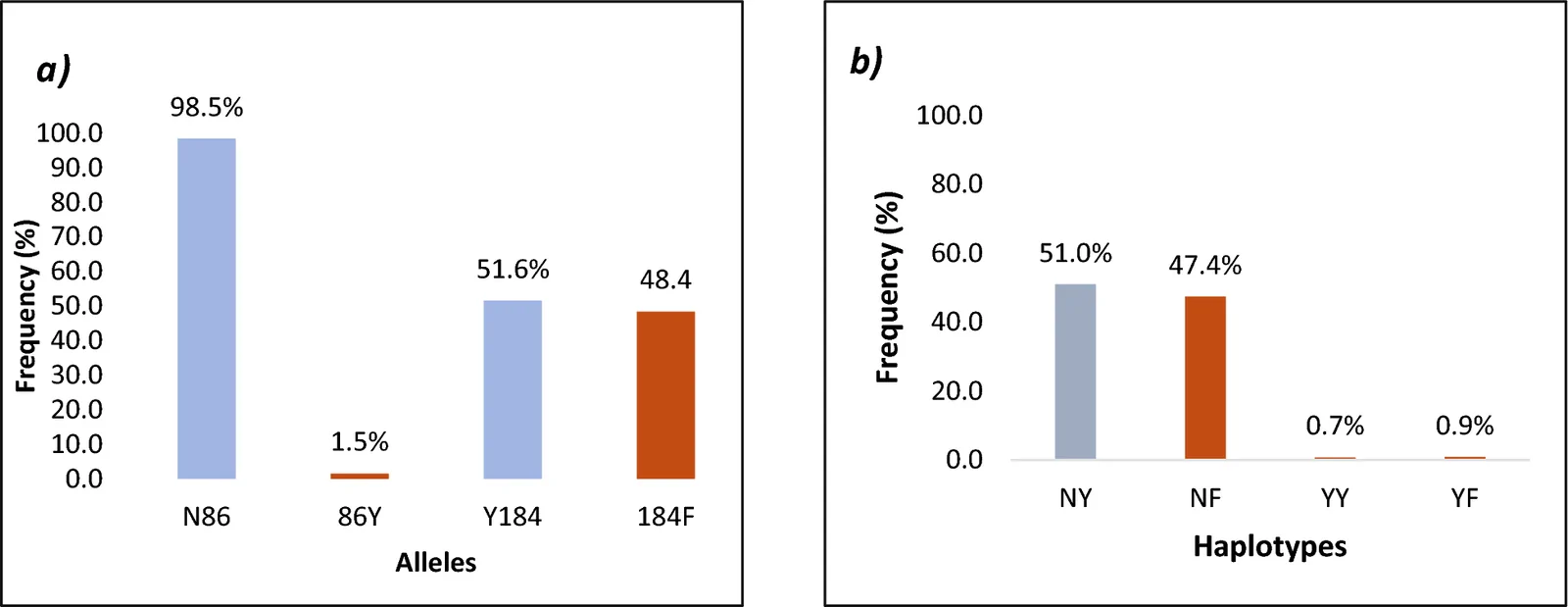
**a**, **b** *Plasmodium falciparum* multidrug Resistance gene 1 codon 86 and 184 a) allele and b) haplotypes frequency. The Allele N86Y was majorly wildtype while Y184F was highly polymorphic. The NY and NF haplotypes were highest

A subset of the 626/675 samples with complete *Pfk13* sequences gave complete readouts of single nucleotide polymorphisms in the *Pfmdr1* gene codon 86 (*n* = 586) and 184 (*n* = 593). SNP frequencies for N86 and 86Y were 98.5% (577/586) and 1.5% (9/586) while those of Y184 and 184F were 51.6% (306/593) and 48.4% (287/593), respectively. 565 samples that had both calls at codons 86 and 184 showed that the *Pfmdr1* 86,184 the NY (51.0%) haplotype was most frequent followed by NF (47.4%), YF (0.9%) and YY (0.7%) (Fig. 4a, b).

### Treatment outcome

Of the individuals 823/927 successfully followed up on day 7 to monitor treatment outcomes, 178/823 (21.6%) had PCR-amplification suggestive of residual parasite nucleic acids, though data for both parasite and drug kinetics was not available. These included 12 samples that, on Day zero, harbored validated artemisinin partial resistance markers *Pfk13* P553L, C469Y, A675V (*n* = 2), and those not previously validated, the V637D mutation. Among the 69 of 178 samples with day-zero SNP data available for both pfmdr1 codons 86 and 184, mutations were detected in 1/69 (2%) and 23/69 (33.3%) of samples at codons 86 and 184, respectively (Table 4). The most frequent haplotype among day-7 positives was *Pfmdr1* N/Y (Fig. 4a, b).

**Table 4:** Parasite residual nucleic acids on day 7 and the *Pfmdr1* 86 and 184 alleles after administering an artemether-lumefantrine dose

Day 0 and 7 samples that amplified for presence of parasite nucleic acids	Day of sample collection	Number of samples (%)
Pfmdr1_86, total	Day 0	71
Pfmdr1_N86	Day 0	66 (93%)
Pfmdr1_86Y	Day 0	1 (1.5%)
Pfmdr1_N86Y	Day 0	2 (2.8%)
Pfmdr1_86, No sequence assembled	Day 0	2 (2.8%)
Pfmdr1_184, total	Day 0	71
Pfmdr1_Y184	Day 0	46 (64.8%)
Pfmdr1_184F	Day 0	18 (25.4%)
Pfmdr1_Y184F	Day 0	5 (7.0%)
Pfmdr1_184, No sequence assembled	Day 0	2 (2.8%)

Parasite nucleic acids detected in comparable proportions in infections that had mutant alleles at day zero, alike to those that were wildtype on day zero

## Discussion

Evolution of mutations in *P. falciparum* Kelch13 propeller domain that is associated with artemisinin partial resistance is on the rise in Africa, sixteen years after the implementation of artemisinin-based combination therapies. The earlier findings [28, 29] showing minimal polymorphisms in the *Pfk13* without clinical implication have been updated by reports of mutations in *Pfk13* C469Y, R561H, A657V, and SNPs mediating delayed parasite clearance after treatment with artemisinin-based combination therapies in East African countries, implying the emergence of resistance in Africa [13, 30]. The current study analyzed a large sample size of 675 parasite isolates collected from symptomatic patients between 2018 and 2024 from seven counties in Kenya for *Pfk13* and other drug resistance mutations. The seven counties represent four of the five distinct malaria transmission zones across Kenya. A total of 24 mutations comprising 13 nonsynonymous, including *Pfk13* A675V (*n* = 20), C469Y (*n* = 3), R561H (*n* = 1) and P553L (*n* = 1), that are on the WHO list of validated partial artemisinin resistance markers previously reported in Africa and Southeast Asia [13, 31, 32], as well as 11 synonymous, were observed across all study sites. This finding seems to indicate that local mutation development, rather than Asian expansion, is driving artemisinin resistance in the African region, based on the parallel reports of these polymorphisms by different studies [13, 31]. Additionally, *Pfmdr1* N86 alongside 184F SNPs were found in 98% and 33.3% of day 0 samples of the infections that tested positive on the day-7 follow-up, respectively.

The global strategy for combating malaria relies on sustained surveillance of drug resistance patterns [33, 34]. The frequency of mutations conferring phenotypic resistance to artemisinin is rising across Africa, posing a public health threat given the weak healthcare infrastructure. This report from a surveillance study in four of the five malaria transmission zones of Kenya, depicting increased genomic activity on *Pfk13,* calls for large-scale surveillance across diverse ecotypes to identify mutations as they arise and estimate their implications on treatment outcome.

The C469Y mutations were reported in one infection from Busia and Kisumu in January 2024. The sample harboring this mutation in Busia also harbored *Pfk13 gene* A675V mutation, which our surveillance had previously reported to be on the rise in Kenya. Three months after this initial detection, a second and third infection in-country, harboring this mutation, was detected in Busia and Kisumu County. Our Busia County referral hospital site is located in Busia town, a major transit town between Kenya and Uganda, connecting Kenya to the Great Lakes region by road. Notably, *Pfk13 gene* C469Y mutation has recently been reported to confer partial artemisinin resistance in Eastern Uganda and Rwanda [7, 13, 35]. The participant in question was five years old, presenting with uncomplicated malaria, and concurrently had a strain harboring the *Pfk13* A675V mutation that is on the rise in Kenya. The participant had parasitemia cleared by day 7 based on malaria rapid diagnostic test and plasmodium genus diagnosis by PCR, suggesting that the participant was successfully treated with artemether-lumefantrine. It is worth noting that this surveillance-type study design, which allowed passive follow-up of participants only, characterized samples from participants who honored the subsequent day 7, 14, 21, 28 and 20 visits and/or unscheduled visits, was unable to document close time-point parasite/drug kinetics during the dosing period that would enable interpretation of the effects of these mutations mimicking the classical therapeutic-efficacy type of studies. 

In October 2023, a study by Jeang and coworkers reported the detection of the *Pfk13 gene* A675V mutation in three field isolates collected from Kakamega County in western Kenya in 2021 [10]. This surveillance study reports a cumulative fivefold increase in the frequency of *Pfk13* A675V mutation between January 2023 and June 2024 from 2/312 (0.6%) to 20/675 (3.0%). The first mutation was detected in Kericho, a highland epidemic malaria zone, in January 2023, and the second one, in Busia in April 2023, about 194 km away but along the same highway. Between October and November 2023, an additional four samples harboring *Pfk13 gene* A675V mutation were detected in Busia (*n* = 1), Kisii (*n* = 1), and Baringo (*n* = 2), bringing the total to 6/476 (1.2%). Between January and February, an additional nine mutations were detected in Busia (*n* = 2), and Baringo (*n* = 7), bringing the overall frequency of the mutation in Kenya to 3.0%. It is worth noting that 60% (12/20) of *Pfk13* A675V mutations were detected in Baringo County, a semi-arid seasonal transmission zone, located over 100 km away from the main road linking Kenya and Uganda [36, 37]. This observation was somewhat new, to the best of our knowledge, in two different ways. First, Kenya is stratified in to five malaria burden zones, placing Baringo County in semi-arid seasonal transmission zone; therefore, inhabitants are inherently less likely to acquire *Plasmodium* species infections per year, hence likely less immunity to malaria as compared to Kisumu and Busia, which are very high burden. Artemisinin partial resistance was expected to follow the classical trend of chloroquine resistance that emerged in holoendemic Kisumu County [38], before spreading and causing epidemics in the less immune individuals in Kisii County. Secondly, only one *Pfk13* mutation, the A675V, was detected in Baringo during the study period. The frequency of infections harboring this mutation increased in Baringo County, faster than in any other county, including those where our surveillance study detected the variant much earlier. Conversely, malaria holoendemic counties had a higher number of SNPs, Busia (*n* = 9), Kisumu (*n* = 8), while those from the highland epidemic zone were few, Kisii (*n* = 4). These observations appear to suggest that the infections in Baringo County were undergoing purifying selection similar to that reported in Southeast Asia, given the low immunity in Baringo County, therefore presenting a unique dilemma to Kenya’s division of the national malaria program’s innovativeness in solving the problem posed by resistant variants in both the immune and less immune population. It is worth noting that the number of participants enrolled per site was derived from individuals who presented at the outpatient department, seeking treatment, providing written informed consent and providing blood samples. The unequal sample sizes are consistent with the differences in malaria transmission and case availability and may affect the precision of site-specific estimates. The WHO has categorized the Pfkelch13 A675V mutation as a validated molecular marker of partial artemisinin resistance based on evidence from studies in East Africa [13, 39]. Though the therapeutic efficacy endpoints were not measured in the present study, the 16.9% frequency of this mutation in Baringo County exceeded the 5% threshold recommended by WHO for classifying areas as regions with suspected partial artemisinin resistance [40]. These findings constitute the first molecular evidence consistent with suspected artemisinin resistance in this Baringo county, highlighting the urgent need for follow-up therapeutic efficacy studies for ascertaining parasite kinetics relative to genomics, parasite clearance dynamics and treatment outcomes [40] Further, these findings warrant deployment of multiple first-line treatments in Baringo County once these are rolled out in Kenya as part of strategies recommended in emerging WHO approaches to resistance management in Africa [41]. Multiple studies from East Africa have documented increasing prevalence of Pfkelch13 mutations associated with artemisinin partial resistance, including A675V in Uganda and Tanzania, as well as R561H and other resistance-associated variants in neighboring countries [9, 35, 39, 42]. The occurrence of A675V in Baringo is therefore consistent with a wider regional pattern of artemisinin resistance emergence across eastern Africa. Based on these findings, Kenya joins other African countries such as Sudan, Ethiopia, Zambia and Namibia that have reported suspected artemisinin resistance [43]. Nevertheless, caution is warranted when interpreting these observations in that, though similar mutations are present across multiple countries [9, 35, 39, 42], the current data do not permit conclusions regarding epidemiological connectivity or cross-border dissemination since it lacks genomic analyses, including haplotype characterization and population genetic studies 

The 13 nonsynonymous and the 11 synonymous mutations included V386A, S600F, K455E, and F491L alongside A626A, A627A, and G690G, respectively; that, to the best of our knowledge, are new. Functional annotation of these mutations revealed that clarifying the role of these mutations in a clinical trial would be worthwhile as a strategy for monitoring the therapeutic efficacy of artemisinin. The P553L mutation was also detected (1%). This mutation, which was previously reported in SEA and Africa, including Kenya [39], is associated with delayed parasite clearance by artemisinin and its derivatives [39, 40] and validated as a marker of artemisinin partial resistance [41–43]. The occurrence of this mutation, among other novel nonsynonymous mutations in Kenyan parasites, is disconcerting and warrants intense surveillance in sub-Saharan Africa and well-designed studies to ascertain their contribution to treatment outcome.

It is noteworthy that all infections resolved based on WHO recommended parasitological diagnosis using the PAN band of the mRDT on day 7. However, 12 of the 44 infections that harbored *Pfk13* mutations on day zero were among the 178 samples that amplified on the day-7 follow-up, depicting likelihood of residual parasite nucleic acids though this surveillance study was not structured to assess parasite and drug kinetics that would ascertain inherent tolerance across different polymorphisms compositions. Pharmacokinetics data was not available to clarify adherence. These 12 samples harbored validated artemisinin partial resistance markers *Pfk13* A578S, P553L, C469Y, A675V (*n* = 2), and those not previously validated V637D–X, N531I A_T, G639R–S, F628L–TT, L571S–F, D559E–G, C437Y - I on day zero.

Synonymous mutations, V637V, V637D, A504A, L488L, C469C, A626A, K455E and G497G were detected, which corroborates earlier findings that SNP V637V exclusively occurs in Africa [41], albeit involving different nucleotides than those found in this investigation. A synonymous mutation C469C (0.3%) was noted in Kisii and Kisumu and likened to the WHO validated marker C469Y [13]. The C469C had previously been detected in Congo [42]. To the best of our knowledge, none of the mixed genotypes discovered by this study, including the synonymous mutations A504A and V386V, have never been reported before. The role of these mutations together with E612D and non-synonymous SNPS F491L in the parasite population before the detection of widespread ACT resistance needs to be investigated in a large sample size.

The large number of polymorphisms reported in this study is evocative of intense molecular events within the *Pfk13,* suggestive of increasing fitness of parasites harboring these mutations in the population. Further, the absence of the mutations that are driving resistance in southeast Asia [3] appears to underscore the role of increased de novo local genetic background of parasite in contributing to parasite fitness, therefore driving susceptibility to drugs. Policy changes are recommended to any ACT regimen in use at the point in time when the frequency of resistance rises above 10% as confirmed using WHO approved clinical efficacy protocols [44]. Therefore, there is an urgent need for early launch of containment efforts as in the Greater Mekong Subregion, but adapted to a greater region of intense transmission in sub-Saharan Africa. Preliminarily, this study was not powered to assess the effect of these polymorphisms on responsiveness to artemisinin derivatives, even though S600F was detected in a day 38 follow-up infection, which had negative results on recrudescence analyses. 

Although we did not collect days 1 and 2 treatment outcome data from patients harboring the newly identified *Pfk13* mutations because this was a passive surveillance study. We encouraged all participants to return on day 7 and any other day within the 42-day follow-up period to monitor treatment outcomes. Hence, only those participants who adhered to the follow-up schedule were characterized. Notably, 21 of the 24 participants who harbored markers of partial artemisinin resistance returned on day 7 with negative microscopy diagnosis. Five of the 24 returned positive PCR diagnosis with late amplifications and could not amplify upon analyses for recrudescence/reinfection analyses, hence not characterized further for recrudescence/reinfections. While this surveillance study design enabled large-scale characterization of infections from diverse locations for the detection of the somewhat low-frequency polymorphisms, it was inherently unable to report on early treatment failures specifically, since the study design emphasized a return for clinical review on day 7, followed by a 42-day follow-up and unscheduled visits.

Non-synonymous SNPs have been implicated in modulating parasite response to drugs and are widely deployed as predictors of emerging resistance or return of susceptibility [45, 46]. Although partial artemisinin resistance has been reported in sub-Saharan Africa and associated with selected *Pfk13* mutations [7, 13, 31, 35, 40], AL is associated with selection of single nucleotide polymorphisms (SNPs) in *P. falciparum* chloroquine resistance transporter gene (*Pfcrt*) and *P. falciparum* multidrug resistance gene 1 (*Pfmdr1*) in parasite re-infections [47].

The lack of in vivo efficacy measurements represents an important limitation: without paired therapeutic response data, we are unable to determine whether these novel variants translate into altered treatment outcomes. Future investigations should integrate structured clinical follow-up, ideally including daily parasite and drug kinetics studies within the first 3 days of treatment, and day 7 and 42-day follow-ups for longer-term cure rates, to fully elucidate the phenotypic relevance of emerging *Pfk13* polymorphisms. We also acknowledge that we did not perform pharmacokinetic measurements to confirm adequate drug exposure. Given that inter‐individual variability in partner‐drug and artemisinin metabolite levels can modulate parasite clearance dynamics, the absence of pharmacokinetics data, tracking drug dynamics alongside parasite kinetics, limits our ability to distinguish between true resistance‐driven delays and sub-therapeutic exposures. Moreover, as multiple studies have demonstrated, the mere presence of *Pfk13* mutations does not invariably predict delayed parasite clearance and conversely, delayed clearance can occur in wild‐type infections, highlighting the need for paired day-3 parasitological and clinical response data alongside both molecular and PK assessments to fully interpret the clinical relevance of detected variants.

## Limitations

Ur surveillance study prioritized high-throughput genomic epidemiology and assessment of day-7 treatment outcomes following Coartem® administration and was therefore not designed to evaluate delayed parasite clearance. Consequently, we did not assess the relationship between Pfk13 mutations and parasitological or clinical treatment outcomes during the critical first three days (days 1–3) after treatment and cannot conclude the impact of these polymorphisms on early therapeutic responses. In addition, annual and site-specific enrollment targets were not consistently achieved because of variations in malaria transmission intensity, seasonality, and case presentation across sentinel sites, resulting in uneven sample sizes across locations and calendar years that may have influenced allele frequency estimates. Despite these limitations, this study provides important insights into the temporal and geographical trends of molecular markers associated with antimalarial drug resistance in P. falciparum across Kenya. The detection of resistance-associated mutations, even at low frequencies, represents an important early warning signal, underscoring the need for sustained molecular surveillance integrated with therapeutic efficacy studies to enable timely detection and containment of emerging artemisinin resistance.

## Conclusion

This surveillance study demonstrates that Plasmodium falciparum Pfk13 polymorphisms associated with partial artemisinin resistance are increasing in Kenya, with a marked rise in the WHO-validated A675V mutation and the emergence of C469Y, R561H, and P553L across multiple malaria transmission zones. The detection of several novel nonsynonymous mutations further appears to suggest ongoing parasite evolution under antimalarial drug pressure. Although this surveillance design was not intended to assess delayed parasite clearance or therapeutic efficacy, the findings provide an important early warning of evolving resistance. These data underscore the need for sustained molecular surveillance integrated with therapeutic efficacy, parasite clearance, and pharmacokinetic studies to determine the clinical significance of emerging variants and to inform timely malaria treatment policy and resistance containment strategies.

## Supplementary Information

Supplementary material 1.

## Data Availability

No datasets were generated or analysed during the current study.

## Code availability

No custom code was generated or used in this study.
