# Supplementary material for: Spatial surveillance of Plasmodium falciparum Kelch 13 mutations reveals High-Frequency A675V and the First Molecular Evidence of Suspected Partial Artemisinin Resistance in Baringo in Kenya
Source: Malar J. 2026 Oct 5;25:389. doi: 10.1186/s12936-026-06102-9 (PMC13721297; doi:10.1186/s12936-026-06102-9)
Supplement: Supplementary file 1 [file 12936_2026_6102_MOESM1_ESM.docx]

| Supplementary Table 1: Single nucleotide polymorphisms amplification | | |
| --- | --- | --- |
|  | **Primer Name** | **Primer sequence** |
| *Pf*K13 | K13F | TGG AAG ACA TCA GTC AAC CAG AGA |
|  | K13R | TTA TAT ATT TGC TAT TAA AAC GGA GTG |
| *Pf*mdr1 | PF86_184 FWD | CCGTTTAAATGTTTACCTGCAC |
|  | PF86_184 RVS | TGGGGTATTGATTCGTTGCAC |
|  | Pfmdr1 1034_1246 F1 | GCATTTAGTTCAGATGATGAAATG |
|  | Pfmdr1 1034_1246 R1 | CCATATGGTCCAACATTTGTATC |
|  |  |  |
| Single nucleotide polymorphisms Sequencing using Sanger Method | | |
|  | **Primer Name** | **Primer sequence** |
| *Pf*K13 | K13F_Seq | GCC AAG CTG CCA TTC ATT TG |
|  | K13R_Seq | GCC TTG TTG AAA GAA GCA GA |
| *Pf*mdr1 | PF86_184 FWD_Seq | GTATGTGCTGTATTATCAGGAG |
|  | PF86_184 RVS_Seq | AGCCTCTTCTATAATGGACATG |
|  | Pfmdr1 1034_1246 F2 | TATGCATACTGTTATTAATTATGG |
|  | Pfmdr1 1034_1246 R2 | TTCGATAAATTCATCTATAGCAG |

**Supplementary Table 2: Parasitemia for samples obtained from seven counties across Kenya**

| County | Number of samples | Minimum | 25% Percentile | Median | 75% Percentile | Maximum |
| --- | --- | --- | --- | --- | --- | --- |
| Laikipia | 2 | 0.04 | 0.04 | 0.12 | 0.2 | 0.2 |
| Kisii | 70 | 0.009 | 0.1 | 0.18 | 0.2525 | 7.9 |
| Busia | 215 | 0.0008 | 0.12 | 0.5 | 1.6 | 16.3 |
| Baringo | 81 | 0.002 | 0.085 | 0.6 | 1.5 | 13 |
| Kericho | 48 | 0.004 | 0.3 | 1.55 | 5.825 | 28.1 |
| Nakuru | 2 | 0.03 | 0.03 | 0.515 | 1 | 1 |
| Kisumu | 323 | 0.00004 | 0.14 | 1.5 | 4 | 22 |

Parastitemia estimated as absolute counts of infected red blood cell per 2000 high power fields. Only samples collected from Laikipia and Nakuru counties. Busia and Baringo had comparable parasitemia, significantly lower that those of Kisumu and Kericho counties.

**Supplementary Table 3: *Pfk13* haplotypes**

| Haplotype | Mutation | Type | Frequency | n |
| --- | --- | --- | --- | --- |
| Haplotype_1 | Wildtype | - | 94.43813 |  |
| Haplotype_2 | A675V  (2024C>T) | Non-synonymous | 2.63503 | 18 |
| Haplotype_3 | C469Y  (1406G>A) | Non-synonymous | 0.29269 | 2 |
| Haplotype_4 | C469Y  (1406G>A)  A675V  (2024C>T) | Non-synonymous  Non-synonymous | 0.14634 | 1 |
| Haplotype_5 | N489K  (1467T>G) | Non-synonymous | 0.14634 | 1 |
| Haplotype_6 | C469C  (1405T>C) | Synonymous | 0.29268 | 2 |
| Haplotype_7 | A675A | Synonymous | 0.14634 | 1 |
| Haplotype_8 | P553L  (1658C>T)  L488L  (1462T>C)  G497G  (1491T>C) | Non-synonymous  Synonymous  Synonymous | 0.14634 | 1 |
| Haplotype_9 | A578S  (1732G>T) | Non-synonymous | 0.5854 | 4 |
| Haplotype_10 | R561H  (1682G>T) | Non-synonymous | 0.14634 | 1 |
| Haplotype_11 | R561P  (1682G>C) | Non-synonymous | 0.14634 | 1 |
| Haplotype_12 | R561R  (1683T>A) | Synonymous |  | 1 |
| Haplotype_13 | R561P  (1682G>C)  R561H  (1682G>T) | Non-synonymous  Non-synonymous |  | 1 |
| Haplotype_14 | A627A  (1881T>A) | Synonymous |  | 1 |
| Haplotype_15 | E612D |  |  | 1 |
| Haplotype_16 | F491L  (1473C>A) | Non-synonymous |  | 1 |
| Haplotype_17 | S600F  (1799C>T) | Non-synonymous |  | 1 |
| Haplotype_18 | A504A  (1512T>C) | Synonymous |  | 1 |
| Haplotype_19 | V637V  (1911T>A) | Synonymous |  | 1 |
| Haplotype_20 | A626A  (1878A>C) | Synonymous |  | 1 |
| Haplotype_21 | K455E  (1363A>G) | Non-synonymous |  | 1 |
| Haplotype_22 | V386A  (1157T>C) | Non-synonymous |  | 1 |
| Haplotype_23 | V386V  (1158T>A)  A675V  (2024C>T) | Synonymous  Non-synonymous |  | 1 |
| Haplotype_24 | A504V  (1511C>T) | Non-synonymous |  | 1 |
| Haplotype_25 | S522C  (1564A>T) | Non-synonymous |  | 1 |
| Haplotype_26 | G690G  (2070C>A) | Synonymous |  | 1 |
|  |  |  |  |  |
